# Association between ANGPTL3, 4, and 8 and lipid and glucose metabolism markers in patients with diabetes

**DOI:** 10.1371/journal.pone.0255147

**Published:** 2021-07-22

**Authors:** Marina Harada, Tadashi Yamakawa, Rie Kashiwagi, Akeo Ohira, Mai Sugiyama, Yasuyuki Sugiura, Yoshinobu Kondo, Yasuo Terauchi

**Affiliations:** 1 Department of Endocrinology and Diabetes, Yokohama City University Medical Center, Yokohama, Japan; 2 Department of Endocrinology and Metabolism, Yokohama City University School of Medicine, Yokohama, Japan; Nagoya University, JAPAN

## Abstract

Lipid management, especially with respect to triglyceride (TG) metabolism, in patients with diabetes is not sufficient with current therapeutic agents, and new approaches for improvement are needed. Members of the angiopoietin-like protein (ANGPTL) family, specifically ANGPTL3, 4, and 8, have been reported as factors that inhibit lipoprotein lipase (LPL) activity and affect TGs. The present study investigated the association between lipid and glucose metabolism markers and the mechanism by which these proteins affect lipid metabolism. A total of 84 patients hospitalized for diabetes treatment were evaluated. Lipid and glucose metabolism markers in blood samples collected before breakfast, on the day after hospitalization, were analyzed. ANGPTL8 showed a significant positive correlation with TG values. HDL-C values displayed a significant positive correlation with ANGPTL3 but a negative correlation with ANGPTL4 and ANGPTL8. The results did not indicate a significant correlation among ANGPTL3, 4, and 8 levels. Thus, it is possible that the distribution of these proteins differs among patients. When patients were divided into groups according to the levels of ANGPTL3 and ANGPTL8, those with high levels of both ANGPTL3 and ANGPTL8 also had high levels of TG and small dense LDL-C/LDL-C (%). Multiple regression analysis indicated that low LPL, high ApoC2, high ApoC3, high ApoE, and high ANGPTL8 levels were the determinants of fasting hypertriglyceridemia. By contrast, no clear association was observed between any of the ANGPTLs and glucose metabolism markers, but ANGPTL8 levels were positively correlated with the levels of HOMA2-IR and BMI. Patients with high levels of both ANGPTL3 and ANGPTL8 had the worst lipid profiles. Among ANGPTL3, 4, and 8, ANGPTL8 is more important as a factor determining plasma TG levels. We anticipate that the results of this research will facilitate potential treatments targeting ANGPTL8 in patients with diabetes.

## Introduction

Dyslipidemia with diabetes is a risk factor for cardiovascular diseases (CVD). It is known that high low-density lipoprotein cholesterol (LDL-C) levels are particularly a strong mediator of dyslipidemia, and the use of LDL-C-lowering drugs [1, 2] including statins, can decrease LDL-C levels and the risk of developing CVD. However, the degree to which high triglyceride (TG) levels and low high-density lipoprotein cholesterol (HDL-C) levels, which are often found in patients with diabetes, contribute to CVD is not as significant as that for high LDL-C levels; nevertheless, at present, management is insufficient. Among patients who manage LDL-C levels within the target range, those with low TG levels were shown to have a low risk of coronary artery disease [3]. This finding highlights the significance of interventions targeting TG levels and the importance of proper management of TG levels as a residual CVD risk. However, several studies on the current TG-lowering drugs, such as fibrate drugs and eicosapentaenoic acid/docosahexaenoic acid (EPA/DHA), report that these drugs do not adequately suppress CVD events, and their positioning as lipid-based therapeutic drugs is not supported by a high level of evidence [4–8]. Further improvements in lipid metabolism, especially TG metabolism, are desired for patients with diabetes and having a high risk of CVD. Therefore, it is necessary to fully elucidate the mechanisms underlying lipid regulation and the actual associated condition in patients with diabetes, in order to establish relevant therapeutic strategies.

Lipoprotein lipase (LPL), a key enzyme that produces free fatty acids (FFAs), is involved in TG degradation [9, 10] and its loss of function causes severe hypertriglyceridemia [9]. LPL hydrolyzes the TGs present in TG-rich lipoproteins, such as chylomicrons (CMs) and very low-density lipoproteins (VLDLs), generates FFAs, and distributes these to peripheral tissues, including the heart [11], muscles [12], and fats [10]. LPL activity appears to be driven via post-translational mechanisms exerted by extracellular proteins. These proteins can be divided into two main groups as follows: liver-derived apolipoproteins (APO)C1, APOC2, APOC3, APOA5, and APOE; and angiopoietin-like protein (ANGPTL)3, ANGPTL4, and ANGPTL8, which have a broader expression profile [10]. Among the eight known ANGPTLs, ANGPTL3, 4, and 8 exert a strong effect on TG metabolism. ANGPTL3 is a circulating factor secreted by, and specifically expressed, in the liver [13]. ANGPTL3 levels are always stable regardless of the nutritional status of the organism [14]. ANGPTL4 specifically inhibits LPL in adipose tissue and modulates LPL activity under fasting- and exercise-related conditions [15]. ANGPTL8 is highly expressed in the liver; its overexpression has been shown to cause a marked increase in TG levels and dose-dependently inhibit LPL activity [16]. Its expression, which is reduced during fasting, is strongly induced by feeding [17]. Furthermore, it inhibits LPL activity in the myocardium and skeletal muscles after feeding [18]. Although plasma TG levels in wild-type mice are elevated when ANGPTL8 is overexpressed, TG levels in *Angptl3*−/− mice are not elevated by overexpression of ANGPTL8 [17]. This suggests that the inhibition of LPL activity by ANGPTL8 depends on ANGTPL3, as opposed to ANGPTL3 and ANGPTL4, which can individually inhibit LPL activity [19]. Thus, all the three ANGPTLs inhibit LPL activity and affect TG metabolism but regulate TG transport via the tissue-specific inhibition of LPL under different nutritional conditions.

Furthermore, ANGPTL4 and ANGPTL8 are associated with glucose metabolism [20]. Elevated ANGPTL4 [21] and ANGPTL8 levels are also associated with obesity [20, 22, 23]. Several clinical studies have indicated significantly elevated serum ANGPTL3, 4, and 8 levels in type 2 diabetes patients compared to healthy controls [24, 25]. However, when such comparisons are limited to patients with type 2 diabetes, there is no significant difference in ANGPTL concentrations between obese and non-obese individuals [24], and neither ANGPTL4 [21] nor ANGPTL8 [26] is associated with glucose metabolism markers. Therefore, the relationship between ANGPTLs and obesity, as well as glucose metabolism, remains controversial due to the different findings pertaining to both diabetes and non-diabetes patients. Furthermore, the results reported by the few studies that investigated the relationship between ANGPTLs and lipid and glucose metabolism in patients with diabetes are inconclusive. Recently, clinical studies have shown improvements in lipid metabolism with an anti-ANGPTL monoclonal antibody [27], and these are attracting attention to develop new therapeutic drugs. In addition, it was shown that ezetimibe, a lipid-improving drug, may lower ANGPTL8 levels [28]; this led to an increased interest in using ANGPTLs as a therapeutic target in clinical practice. The purpose of this study was thus to clarify the interactions among circulating ANGPTL3, 4, and 8 levels, their association with lipid and glucose metabolism markers, and the contributing factors to plasma TG levels in patients with diabetes. We anticipate that the results of this research will lead to the advent of new potential treatments targeting ANGPTLs in patients with diabetes.

## Materials and methods

### Ethical considerations and subjects

This was a prospective observational study conducted at a single institution for patients with diabetes, admitted for the purpose of controlling blood glucose levels. This study complied with the provisions of the Declaration of Helsinki and was approved by the Yokohama City University Hospital Ethics Committee (approval number: B170700048) to start in September 2017. The study was registered in the UMIN-CTR in September 2018 (UMIN000034317) because the applicant was not registered in the UMIN-CTR. All the patients provided written informed consent. We report and submit a portion of the data and results from this study. Patients with uncontrolled diabetes over the age of 20 years who were admitted to the Department of Endocrinology and Diabetes, Yokohama City University Medical Center, between November 2017 and March 2019 were selected. Exclusion criteria were as follows: (i) severe ketosis; (ii) diabetic coma; (ⅲ) serious infection; and (iv) serious trauma. Of the 113 patients who participated in the study, 29 patients with cancer as comorbidity were excluded, and the remaining 84 were further analyzed. Of these, five were type 1 diabetes patients and 79 were type 2 diabetes patients (protocol: https://doi.org/10.17504/protocols.io.bmjsk4ne).

### Clinical evaluation and laboratory measurements

Blood sampling was performed early in the morning for patients that had fasted for at least 12 h, on the day after admission, following which samples were immediately centrifuged at 3000 rpm for 5 min. Next, samples required for the measurement of GPIHBP1, HTGL, ANGPTL3, ANGPTL4, ANGPTL8, and lipoprotein subclasses were frozen at −30°C and stored. GPIHBP1 was measured by the sandwich method using a Human GPIHBP1 Enzyme linked Immunosorbent Assay (ELISA) Kit (#27179 Human GPIHBP1 Assay Kit—IBL; Immuno-Biological Laboratories [IBL], Gunma, Japan). HTGL was also measured using the Human Serum HTGL ELISA Kit (#27180 Human Serum HTGL Assay Kit—IBL). LPL was measured without the intravenous injection of heparin. Only HbA1c values were obtained within 1 month of hospitalization. Beta cell function (HOMA2-%β) and insulin resistance (HOMA2-IR) were calculated via a HOMA Calculator using fasting plasma glucose levels and C-peptides (https://www.dtu.ox.ac.uk/homacalculator/download.php).

The patient characteristics were defined as follows: neuropathy, the presence of a subjective symptom (palsy, pain, etc.) or the weakening of Achilles tendon reflex, sense of vibration, or sensory nerve conduction velocity; nephropathy, diabetic nephropathy stages 2, 3, 4, or 5; retinopathy, simple, preproliferative, or proliferative diabetic retinopathy; macroangiopathy, ischemic heart disease, cerebral infarction, and arteriosclerosis obliterans.

### Measurement of ANGPTL3, 4, and 8 levels

Blood samples used to measure ANGPTL3, 4, and 8 levels were first centrifuged immediately after blood collection and stored at 4°C; after extracting only plasma components within 2 d, the samples were stored at −30°C in the hospital. The samples were transported to IBL in a frozen state, which required thawing twice before conducting measurements due to the division of the samples. ANGPTL3 was measured using a Human ANGPTL3 ELISA Kit (#27750 Human ANGPTL3 [highly sensitive] Assay Kit—IBL) via the sandwich method, and similarly, ANGPTL4 was measured using a Human ANGPTL4 ELISA Kit (#27749 Human ANGPTL4 Assay Kit—IBL). ANGPTL8 was measured using a Human ANGPTL8 ELISA Kit (#27795 Human ANGPTL8 Assay Kit—IBL). The specificity of the ANGPTL3, 4, and 8 antibodies was confirmed, where cross-reactivity was less than 0.1%.

### Lipoprotein analysis

Lipoprotein subclasses were measured via gel-permeation high-performance liquid chromatography (GP-HPLC; LipoSERCH, Skylight Biotech, Inc., Tokyo, Japan), which facilitates the measurement of four major lipoprotein classes and 20 detailed lipoprotein subclasses of total cholesterol (TC) and TG. The former were classified as CM, VLDL, LDL-C, and HDL-C, whereas the latter were classified using particle diameter as follows: CM subclass 1–2; VLDL subclass 1–5; LDL-C subclass 1–6; and HDL-C subclass 1–7. The six LDL-C subclasses are large LDL-C (LDL-1, 28.6 nm in diameter), medium LDL-C (LDL-2, 25.5 nm), small LDL-C (LDL-3, 23 nm), and very small LDL-C (LDL4–6, 20.7–16.7 nm). In this study, we defined LDL-C subclass 3–6 (23–16.7 nm) as small dense (sd) LDL-C, and LDL-C subclass 1–2 (28.6–25.5 nm) as large buoyant (lb) LDL-C. CM-TG, VLDL-TG, and LDL-TG were measured using GP-HPLC (LipoSERCH, Skylight Biotech), and then, TG content in CM, VLDL, and LDL-C was evaluated.

### Statistical analysis

HOMA2-%β, HOMA2-IR, TG, LPL, GPIHBP1, THGL, ApoC2, ApoC3, ApoE, ANGPTL3, ANGPTL4, ANGPTL8, CM-TG, VLDL-TG, and LDL-TG were transformed to natural-log values to enable statistical analyses, as the frequency distributions of these variables were found to be skewed. The results were expressed as either mean (± standard deviation), median (25–75% inter-quartile range), or numbers with percentages. Comparisons among the four categories were performed via Tukey’s multiple comparison test, where the results were adjusted for BMI and expressed as mean (± standard error). To identify factors that were independently correlated with plasma TG levels, univariate and multivariate linear regression analyses were conducted. Univariate analysis was first conducted, and variables, such as BMI, LPL, ApoC2, ApoC3, ApoE, ANGPTL8, GPIHBP1, and HTGL, which satisfied the criterion of P < 0.05, were collectively entered in multivariate analyses, with ANGPTL3 and ANGPTL4 as factors that affect TG levels. In addition, the standard partial regression coefficient (β) is shown to compare the degree to which each explanatory variable affects the TG values. All independent variables in the multivariate linear regression analyses were tested for multicollinearity to ensure that variance inflation factor did not exceed 5–10. Two-tailed P-values < 0.05 were considered statistically significant. Statistical analyses were performed using JMP Pro 12.0.0 software (SAS Institute, Cary, NC).

## Results

### General characteristics

Baseline characteristics of the 84 patients are presented in Table 1. The parameters of lipid and glucose metabolism are shown in Table 2. Mean HbA1c was 10.1 ± 2.1%, indicating poor glycemic control. The values of TG, LDL-C, and HDL-C were appropriately controlled.

**Table 1:** Baseline characteristics.

Age (years)	62.9 ± 14.4
Sex (male/female)	57/27
Body weight (kg)	69.0 ± 15.8
BMI (kg/m^2^)	25.9 ± 4.5
Estimated duration (years)	10 (5–10)
Current smoker (%)	22.6
Hypoglycemic treatment	
OHA only (%)	60.7
Insulin or GLP-1 (%)	31
None (%)	8.3
Hypolipidemic treatment	
Statin (%)	42.9
Non-statin (%)	8.3
None (%)	48.8
Neuropathy (%)	65.4
Nephropathy (%)	33.3
Retinopathy (%)	32
Macroangiopathy (%)	17.9

Values are expressed as mean ± standard deviation, median (25–75% inter-quartile range), or percentage (%). BMI, body mass index; OHA, oral hypoglycemic agent; GLP-1, Glucagon-like peptide-1.

**Table 2:** Clinical characteristics and laboratory data.

HbA1c (%)	10.1 ± 2.1
FPG (mg/dL)	155 (132–184)
C-peptide (ng/mL)	1.9 (1.1–2.6)
HOMA2-%β	101.4 (73–141.2)
HOMA2-IR	4.7 (2.6–7.1)
AST (U/L)	19 (15–25)
ALT (U/L)	18 (13–27)
γGTP (U/L)	28.5 (18–50)
eGFR (mL/min/1.73 m^2^)	71.1 ± 19.9
TG (mg/dL)	123 (94.3–173.3)
Total cholesterol (mg/dL)	194.5 ± 42.8
LDL-C (mg/dL)	114.8 ± 33.1
HDL-C (mg/dL)	48.6 ± 11.2
non-HDL-C (mg/dL)	141.5 (118.3–170.8)
LPL (ng/mL)	44 (34–61)
GPIHBP1 (pg/mL)	990 (821.8–1173.5)
HTGL (ng/mL)	54.8 (42.6–73.7)
ApoA1 (mg/dL)	138.5 ± 22.4
ApoA2 (mg/dL)	27.6 ± 5.2
ApoB (mg/dL)	98.8 ± 25.3
ApoC2 (mg/dL)	4.8 (3.9–7.3)
ApoC3 (mg/dL)	10.2 (8.2–14)
ApoE (mg/dL)	4.3 (3.6–5.6)
CM-TG (mg/dL)	9.49 (4.1–21.5)
VLDL-TG (mg/dL)	84.8 (57.6–126.0)
LDL-TG (mg/dL)	27.5 (21.5–34.1)
sd LDL-C (mg/dL)	24.3 (16.7–32.7)
lb LDL-C (mg/dL)	71.2 ± 20.9
sd LDL-C/LDL-C (%)	26.7 ± 6.5
ANGPTL3 (ng/mL)	349.7 (272.7–429)
ANGPTL4 (pg/mL)	241.9 (156.2–381.8)
ANGPTL8 (pmol/L)	49.8 (33.3–75.7)

Values are expressed as mean ± standard deviation or median (25–75% inter-quartile range). HbA1c, glycated hemoglobin; FPG, fasting plasma glucose; AST, aspartate transaminase; ALT, alanine transaminase; γGTP, γ-glutamyl transpeptidase; eGFR, estimated glomerular filtration rate; TG, triglycerides; LDL-C, low-density lipoprotein cholesterol; HDL-C, high-density lipoprotein cholesterol; LPL, lipoprotein lipase; GPIHBP1, glycosylphosphatidylinositol anchored high-density lipoprotein binding protein 1; HTGL, hepatic triacylglycerol lipase; Apo, apolipoproteins; CM, chylomicron; VLDL, very low-density lipoproteins; sd LDL-C, small dense LDL-C; lb LDL-C, large buoyant LDL-C; ANGPTL, angiopoietin-like protein. Values of HOMA2-%β and HOMA2-IR were calculated via the HOMA Calculator using fasting plasma glucose levels and C-peptides (https://www.dtu.ox.ac.uk/homacalculator/download.php).

### Association between lipid and glucose metabolism parameters and ANGPTL3, 4, and 8

To evaluate the potential association between ANGPTL levels and glucose, as well as lipid markers, we conducted univariate regression analysis. ANGPTL3 showed a positive correlation with HDL-C and ApoA1 levels (Table 3). ANGPTL4 levels showed a negative correlation with HDL-C and a positive correlation with ApoC2 levels (Table 3). ANGPTL8 levels were significantly higher with respect to categories such as BMI, HOMA2-%β, HOMA2-IR, TG, GPIHBP1, ApoC2, ApoC3, and ApoE, and they were negatively correlated with HDL-C (Table 3). Our results did not indicate a significant correlation between ANGPTL3, 4, and 8 levels; however, a clear association was observed between the markers of lipid and glucose metabolism and ANGPTL levels.

**Table 3:** Association between lipid and glucose parameters and ANGPTL3, 4, and 8.

	log (ANGPTL3)			log (ANGPTL4)			log (ANGPTL8)		
	B	95% CI	P	B	95% CI	P	B	95% CI	P
Age	0.003	(-0.002, 0.01)	0.224	-0.01	(-0.02, 0.002)	0.102	0.003	(-0.007, 0.013)	0.572
Sex	-0.058	(-0.215, 0.099)	0.461	0.624	(0.345, 0.902)	< 0.0001	-0.191	(-0.489, 0.108)	0.207
HbA1c	0.0001	(-0.035, 0.035)	0.997	0.019	(-0.05, 0.89)	0.586	-0.029	(-0.096, 0.038)	0.389
FPG	-0.0002	(-0.002, 0.002)	0.848	-0.0004	(-0.004, 0.003)	0.806	0.001	(-0.002, 0.004)	0.499
BMI	-0.021	(-0.037, -0.01)	0.008	0.03	(-0.002, 0.06)	0.065	0.064	(0.036, 0.091)	< 0.0001
log (HOMA2-%β)	-0.04	(-0.131, 0.053)	0.4	0.129	(-0.053, 0.311)	0.161	0.297	(0.13, 0.463)	0.001
log (HOMA2-IR)	-0.031	(-0.152, 0.9)	0.611	0.165	(-0.072, 0.402)	0.169	0.498	(0.293, 0.703)	< 0.0001
log (TG)	-0.054	(-0.208, 0.101)	0.493	0.17	(-0.133, 0.473)	0.269	0.668	(0.411, 0.926)	< 0.0001
LDL-C	0.0006	(-0.002, 0.003)	0.593	0.003	(-0.002, 0.007)	0.211	-0.0002	(-0.004, 0.004)	0.936
HDL-C	0.01	(0.004, 0.016)	0.0015	-0.017	(-0.03, -0.004)	0.01	-0.016	(-0.03, -0.004)	0.011
Non-HDL-C	0.0004	(-0.001, 0.002)	0.659	0.002	(-0.002, 0.005)	0.335	0.002	(-0.001, 0.005)	0.236
log (LPL)	0.154	(-0.059, 0.367)	0.367	-0.34	(-0.758, 0.078)	0.109	0.034	(-0.379, 0.447)	0.871
log(GPIHBP1)	0.004	(-0.22, 0.229)	0.97	0.137	(-0.306, 0.579)	0.541	0.675	(0.271, 1.079)	0.001
log (HTGL)	0.01	(-0.168, 0.187)	0.915	0.183	(-0.165, 0.531)	0.298	0.295	(-0.038, 0.629)	0.082
ApoA1	0.004	(0.002, 0.008)	0.0032	-0.005	(-0.011, 0.002)	0.134	-0.003	(-0.01, 0.003)	0.305
ApoA2	0.01	(-0.005, 0.024)	0.184	0.037	(0.01, 0.063)	0.008	0.009	(-0.018, 0.036)	0.494
ApoB	0.0001	(-0.003, 0.003)	0.912	0.005	(-0.001, 0.01)	0.117	0.004	(-0.001, 0.01)	0.135
log (ApoC2)	-0.046	(-0.209, 0.117)	0.578	0.243	(-0.075, 0.561)	0.132	0.382	(0.08, 0.683)	0.014
log (ApoC3)	0.01	(-0.168, 0.189)	0.908	-0.032	(-0.384, 0.319)	0.856	0.591	(0.276, 0.907)	0.0004
log (ApoE)	0.216	(-0.024, 0.456)	0.077	0.036	(-0.446, 0.518)	0.883	0.861	(0.433, 1.289)	0.0001
log (ANGPTL4)	0.17	(-0.23, 0.6)	0.426				0.011	(-0.215, 0.237)	0.923
log (ANGPTL8)	-0.329	(-0.74, 0.08)	0.117						

A generalized linear model was used to investigate the relationship between ANGPTLs and clinical parameters. Sex: male = 1, female = 0. B, partial regression coefficient; 95% CI, 95% confidence interval; P, probability; HbA1c, glycated hemoglobin; FPG, fasting plasma glucose; BMI, body mass index; TG, triglyceride; LDL-C, low-density lipoprotein cholesterol; HDL-C, high-density lipoprotein cholesterol; LPL, lipoprotein lipase; GPIHBP1, glycosylphosphatidylinositol anchored high-density lipoprotein binding protein 1; HTGL, hepatic triacylglycerol lipase; Apo, apolipoproteins; ANGPTL, angiopoietin-like protein. Values of HOMA2-%β and HOMA2-IR were calculated via the HOMA Calculator using fasting plasma glucose levels and C-peptides (https://www.dtu.ox.ac.uk/homacalculator/download.php).

### Patients with high concentrations of both ANGPTL3 and ANGPTL8 exhibit the worst lipid profiles

**Fig 1:**
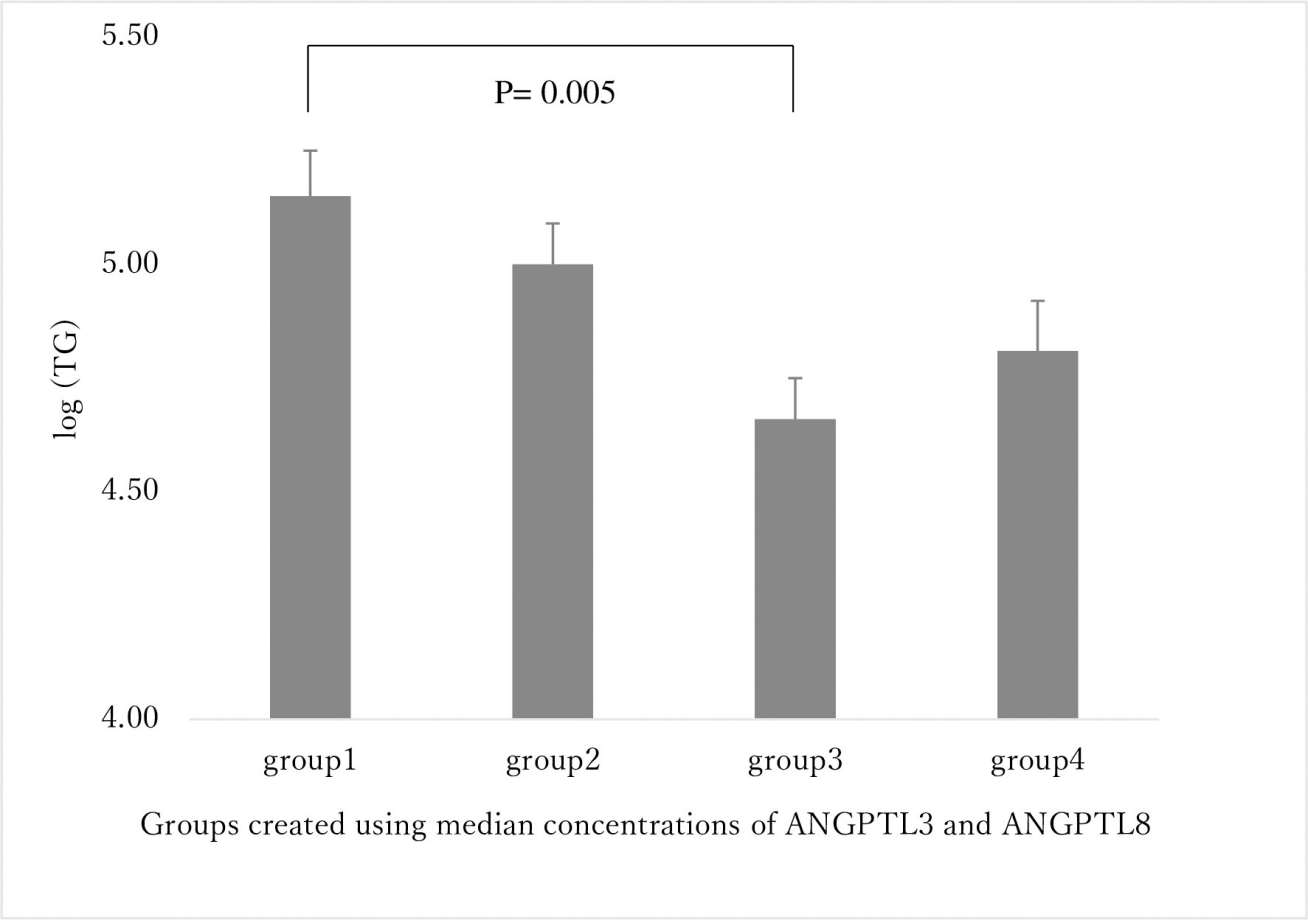
The relationship between TG levels and the four groups created using the median concentrations of ANGPTL3 and ANGPTL8. Subjects were divided into four groups based on the median concentrations of ANGPTL3 and ANGPTL8 as follows: group 1, both ANGPTL3 and ANGPTL8 concentrations were higher than the median (ANGPTL3 ≥ 349.7 ng/mL, ANGPTL8 ≥ 49.8 pmol/L); group 2, the concentration of ANGPTL3 was below the median and that of ANGPTL8 was above the median (ANGPTL3 < 349.7 ng/mL, ANGPTL8 ≥ 49.8 pmol/L); group 3, the concentration of ANGPTL3 was above the median and that of ANGPTL8 was below the median (ANGPTL3 ≥ 349.7 ng/mL, ANGPTL8 < 49.8 pmol/L); and group 4, both ANGPTL3 and ANGPTL8 concentrations were below the median (ANGPTL3 < 349.7 ng/mL, ANGPTL8 < 49.8 pmol/L). Comparisons among the four groups were performed via Tukey’s multiple comparison test after adjusting for BMI. TG, triglycerides; ANGPTL, angiopoietin-like protein.

ANGPTL8 exerts its LPL inhibitory effect only in the presence of ANGPTL3. To investigate the effects of ANGPTL3 and 8 on lipid parameters, we divided the subjects into four groups: (1) both ANGPTL3 and ANGPTL8 higher than the median (ANGPTL3 ≥ 349.7 ng/mL, ANGPTL8 ≥ 49.8 pmol/L); (2) ANGPTL3 below the median and ANGPTL8 above the median (ANGPTL3 < 349.7 ng/mL, ANGPTL8 ≥ 49.8 pmol/L); (3) ANGPTL3 above the median and ANGPTL8 below the median (ANGPTL3 ≥ 349.7 ng/mL, ANGPTL8 < 49.8 pmol/L); and (4) both ANGPTL3 and ANGPTL8 below the median (ANGPTL3 < 349.7 ng/mL, ANGPTL8 < 49.8 pmol/L). The correlations between these four groups and lipid parameters are shown, after adjusting for BMI (Fig 1 and Table 4). The log (TG) value was significantly higher in group 1 (5.2 ± 0.1) than in group 3 (p = 0.005; Fig 1). Next, we compared the TG concentrations of the lipoproteins CM-TG, VLDL-TG, and LDL-TG and investigated the qualitative features of LDL particles such as sd LDL-C and lb LDL-C. The log (CM-TG) of group 1 was significantly higher than that of groups 2 and 3 (2.9 ± 0.2, versus group 2, group 3; P < 0.05), whereas the log (VLDL-TG) was also significantly higher in group 1 (4.7 ± 0.1, versus group 2, group 3; P < 0.05; Table 4). However, there was no significant difference among groups regarding log (LDL-TG), sd LDL-C, and lb LDL-C values, but sd LDL-C/LDL-C (%) was significantly higher in group 1 (29.3 ± 1.5, group 3, P < 0.05; Table 4).

**Table 4:** The relationship between lipid profiles and the four groups created using the median concentrations of ANGPTL3 and ANGPTL8.

	Group 1	Group 2	Group 3	Group 4
n	17	26	25	16
log (CM-TG)	2.9 ± 0.2	2.5 ± 0.2	1.7 ± 0.2^†‡^	2.2 ± 0.2
log (VLDL-TG)	4.7 ± 0.1	4.6 ± 0.1	4.2 ± 0.1^†‡^	4.3 ± 0.1
log (LDL-TG)	3.4 ± 0.1	3.3 ± 0.1	3.4 ± 0.1	3.3 ± 0.1
sd LDL-C (mg/dl)	27.2 ± 3.2	28.3 ± 2.7	25.9 ± 2.8	25.1 ± 3.3
lb LDL-C (mg/dl)	64.3± 5	68.4 ± 4.2	80.4 ± 4.3	68.9 ± 5.2
sd LDL/LDL-C (%)	29.3 ± 1.5	27.9 ± 1.3	24.1 ± 1.3^†^	26.3 ± 1.5

Subjects were divided into four groups based on the median concentrations of ANGPTL3 and ANGPTL8 as follows: group 1, both ANGPTL3 and ANGPTL8 concentrations were higher than the median (ANGPTL3 ≥ 349.7 ng/mL, ANGPTL8 ≥ 49.8 pmol/L); group 2, the concentration of ANGPTL3 was below the median and that of ANGPTL8 was above the median (ANGPTL3 < 349.7 ng/mL, ANGPTL8 ≥ 49.8 pmol/L); group 3, the concentration of ANGPTL3 was above the median and that of ANGPTL8 was below the median (ANGPTL3 ≥ 349.7 ng/mL, ANGPTL8 < 49.8 pmol/L); and group 4, both ANGPTL3 and ANGPTL8 concentrations were below the median (ANGPTL3 < 349.7 ng/mL, ANGPTL8 < 49.8 pmol/L). Comparisons among the four categories were performed via Tukey’s multiple comparison test after adjusting for BMI; the results are expressed as mean ± standard error. CM, chylomicron; TG, triglycerides; VLDL, very low-density lipoproteins; sd LDL-C, small dense LDL-C; lb LDL-C, large buoyant LDL-C; ANGPTL, angiopoietin-like protein.

†P < 0.05 versus group 1, ‡versus group 2.

### High ANGPTL8 levels have a significant impact on high TG levels

Multiple regression analysis was conducted to elucidate factors that influence fasting TG levels and to clarify how ANGPTLs affect plasma TG levels. The results revealed that low LPL levels and high ApoC2, ApoC3, ApoE, and ANGPTL8 values were variables that affected plasma TG levels (Table 5).

**Table 5:** Univariate and multivariate linear regression analyses of plasma TG levels and factors affecting TGs.

	Univariate			Multivariate			
	B	95% CI	P	B	95% CI	β	P
Age	-0.006	(-0.01, 0.002)	0.132				
Sex	0.144	(-0.08, 0.37)	0.197				
BMI	0.033	(0.01, 0.055)	0.004	-0.005	(-0.02, 0.007)	-0.045	0.419
HbA1c	0.016	(-0.034, 0.07)	0.535				
log (LPL)	-0.447	(-0.74, -0.16)	0.003	-0.264	(-0.39, -0.134)	-0.191	0.0001
log (GPIHBP1)	0.541	(0.245, 0.837)	0.001	0.085	(-0.06, 0.227)	0.059	0.242
log (HTGL)	0.399	(0.164, 0.636)	0.001	0.016	(-0.095,0.128)	0.014	0.774
log (ApoC2)	0.842	(0.703, 0.982)	< 0.0001	0.198	(0.018, 0.378)	0.188	0.032
log (ApoC3)	0.993	(0.866, 1.121)	< 0.0001	0.538	(0.32, 0.755)	0.467	< 0.0001
log (ApoE)	1.152	(0.915, 1.389)	< 0.0001	0.276	(0.045, 0.508)	0.175	0.02
log (ANGPTL3)	-0.108	(-0.42, 0.203)	0.493	-0.088	(-0.23, 0.06)	-0.062	0.232
log (ANGPTL4)	0.088	(-0.07, 0.245)	0.269	0.051	(-0.02, 0.121)	0.071	0.154
log (ANGPTL8)	0.367	(0.225, 0.508)	< 0.0001	0.137	(0.052, 0.222)	0.186	0.002

Sex: male = 1, female = 0. B, partial regression coefficient; 95% CI, 95% confidence interval; β, standardized partial regression coefficient; P, probability; TG, triglycerides; BMI, body mass index; HbA1c, glycated hemoglobin; LPL, lipoprotein lipase; GPIHBP1, glycosylphosphatidylinositol anchored high-density lipoprotein binding protein 1; HTGL, hepatic triacylglycerol lipase; Apo, apolipoproteins; ANGPTL, angiopoietin-like protein.

## Discussion

### Summary

We investigated the correlation among circulating ANGPTL3, 4, and 8 levels and the association between ANGPTLs and markers of lipids, as well as glucose metabolism, in patients with diabetes. No significant correlation was observed among the circulating levels of these proteins. Regarding the correlation with lipid metabolism, circulating ANGPTL8 levels were positively correlated with TG levels, whereas circulating ANGPTL3 levels were positively correlated with HDL-C levels, while ANGPTL4 and ANGPTL8 levels were negatively correlated with HDL-C levels. However, no significant correlation was observed between HbA1c or glucose levels and these proteins, although ANGPTL8 levels were positively correlated with HOMA2-IR and BMI values. Furthermore, patients were divided into groups according to the concentrations of plasma ANGPTL3 and ANGPTL8 levels to investigate their lipid profile. The group in which both the ANGPTL3 and ANGPTL8 levels were high, showed the worst lipid profile pertaining to increased TG levels and sd LDL ratios. Multiple regression analyses indicated that ANGPTL8 levels were important factors related to fasting hypertriglyceridemia in diabetes patients.

### Relationship between ANGPTL3, 4, and 8 and lipid metabolism

The present study showed that ANGPTL8 displayed a significant, positive correlation with fasting TG levels, whereas ANGPTL3 did not. A recent study proposed that a significant increase in fasting plasma TG levels observed in mice overexpressing ANGPTL8 [19] was caused by fasting-induced inhibition of LPL in both adipocytes and skeletal muscles which, in turn, led to a significant elevation of plasma TG levels in the presence of excess ANGPTL8 [14]. We surmised that high ANGPTL8 levels may induce hypertriglyceridemia in a fasting state, which is consistent with the findings of a study conducted by Haller et al. [19]. The study indicated that ANGPTL8 induces major LPL inhibitory activity when ANGPTL3 and ANGPTL8 are co-expressed. For other lipoproteins, ANGPTL3 levels were positively correlated with HDL-C levels, probably because ANGPTL3 increases HDL-C levels by inhibiting endothelial lipase [27, 29–31]. However, ANGPTL4 and ANGPTL8 levels were negatively correlated with HDL-C levels, which is consistent with the previous reports that HDL-C levels are increased by ANGPTL4 deficiency [32, 33]. HDL-C levels were increased by low ANGPTL8 levels [34], probably because LPL inhibition increased FFA and promoted hepatic VLDL secretion. Our study showed no significant association between the levels of ANGPTL3, 4, and 8 and LDL-C levels. Reportedly, LDL-C levels in humans are reduced due to a lack of ANGPTL3 [27, 31], although the reason for this remains unclear [35]. It was reported that LDL-C levels remain unchanged following the administering of ANGPTL8 human monoclonal antibodies to mice [34]; however, another study indicated that LDL-C levels decline in humans lacking ANGPTL8 [17], demonstrating inconsistencies in such results, the reasons for which remain unknown.

### Interaction of ANGPTLs

Circulating ANGPTL8 levels appear to affect TG levels more than ANGPTL3. We focused on the interaction between ANGPTL3, 4, and 8 to examine the underlying mechanisms. ANGPTL3 increases plasma FFA via lipolysis, and increasing FFA in turn upregulates ANGPTL4 via the activation of peroxisome proliferator-activated receptor (PPAR)-γ/α/δ [36]. ANGPTL3 and ANGPTL8 regulate LPL activity via an interaction. Thus, ANGPTL3, 4, and 8 jointly regulate LPL activity. However, our plasma concentration measurements showed no significant correlation among these proteins, which was consistent with the results of other clinical studies [20, 24]. Although the individual distributions of all the three ANGPTLs are different, these proteins, which are intricately regulated, appear to inhibit LPL activity and cause hypertriglyceridemia, the dynamics of which cannot be explained via a central dogma.

### Groups with high levels of both ANGPTL3 and ANGPTL8 exhibit the worst lipid profile, indicating that circulating ANGPTL8 levels are significant determinants of plasma TG levels

We focused on the observation that ANGPTL3 is required by ANGPTL8 to inhibit LPL [14, 17, 19] and the co-expression of ANGPTL3 and ANGPTL8 induces more pronounced hypertriglyceridemia than the overexpression of ANGPTL3 alone [17, 19]. Furthermore, the correlations between plasma TG levels and the concentrations of these proteins were investigated. Results indicated that the TG levels were significantly higher in patients with high plasma levels of both ANGPTL3 and ANGPTL8. Moreover, sd LDL-C/LDL-C (%) was significantly higher in this group. These results indicated that the lipid profile of the group with both higher ANGPTL3 and ANGPTL8 was poor. However, which ANGPTL—ANGPTL3, 4, or 8—exerts the largest effect on the plasma TG levels of diabetes patients has not been investigated thus far. Multiple regression analyses suggested that ANGPTL8 among ANGPTL3, 4, and 8, plays an important role in determining plasma TG levels. Morinaga et al. [20] reported that the interaction between ANGPTL3 and ANGPTL8 is positively correlated with increased circulating TG levels, although the effect was less than that of circulating ANGPTL8 levels alone. Haller et al. [19] suggested that the ANGPTL8 "inhibitory motif" is required to inhibit LPL when ANGPTL8 is co-expressed with ANGPTL3. Moreover, the increase in plasma TG levels was found to be enhanced in response to the co-expression of ANGPTL3 and ANGPTL8, despite a much lower level of circulating ANGPTL3. Using mass spectrometry, Chen et al. [37] showed that ANGPTL8 is mostly present in the blood, not free but forming a complex with other ANGPTLs. This suggests that when plasma ANGPTL8 levels are high, the ANGPTL3/8 complex is also abundant in the blood. Our study measured ANGPTLs by ELISA and could not distinguish whether ANGPTLs were free or complexed. However, our overall findings, that high ANGPTL8 levels contribute to TG elevation, suggest that the IBL-ELISA ANGPTL8 measures complexed ANGPTL8. On the other hand, they also revealed that many ANGPTL3 do not form a complex but exist in a free state. In addition, LPL activity inhibition of the ANGPTL3/8 complex was more than 100 times stronger than that of free ANGPTL3. This further supports our findings, as high ANGPTL8 levels are thought to contribute to hypertriglyceridemia rather than high ANGPTL3 levels. It is anticipated that future research will reveal the reasons behind the display of a stronger LPL inhibitory effect by ANGPTL8 in the presence of ANGPTL3, as well as the underlying mechanism.

### Relationship between ANGPTL3, 4, and 8 and glucose metabolism

ANGPTL3 deficiency improves insulin sensitivity in both humans and mice [38, 39]. Experimental results have consistently indicated an association between ANGPTL3 and glucose metabolism. Previous studies clearly demonstrated that the pathology associated with excess ANGPTL3 causes impaired glucose tolerance and increases insulin resistance in healthy individuals. However, our study suggests that a significant correlation between ANGPTL3 levels and glucose metabolism markers is lacking in patients with diabetes. Mice overexpressing ANGPTL4 reportedly show impaired glucose tolerance [40, 41], and conversely, ANGPTL4-deficient mice show improved glucose tolerance [42]. Although ANGPTL4 is reportedly elevated in patients with impaired glucose tolerance and obesity, no significant association was observed between ANGPTL4 and HOMA-IR, HbA1c, or BMI values in patients with impaired glucose tolerance, including those with type 2 diabetes [21, 24]. The present study, which monitored diabetes patients, revealed that a significant correlation among obesity, glucose metabolism markers, and ANGPTL4 is lacking. ANGPTL8 reportedly plays an important role in TG metabolism without impairing glucose homeostasis in mice [43, 44]. However, some clinical reports have demonstrated that circulating ANGPTL8 levels in type 2 diabetes patients are elevated compared with those in non-diabetes individuals [23–26]; ANGPTL8 levels are also elevated in obese individuals [20, 22, 23, 25]. Our study also showed the absence of a significant association between ANGPTL8 levels and HbA1c or glucose levels. This revelation was similar to that of a report indicating that ANGPTL8 levels are not associated with glucose metabolism markers in patients with type 2 diabetes [26]. In contrast, we revealed that ANGPTL8 levels have a significantly positive correlation with BMI and HOMA2-IR values, which suggests that it may be associated with obesity and insulin resistance in patients with diabetes.

### Future outlook

The present study found that the circulating ANGPTL8 levels contribute to elevated TG levels and may also be associated with obesity and insulin resistance in patients with diabetes. This suggests that interventions that reduce circulating ANGPTL8 levels can improve lipid profiles in diabetes patients with poor TG control and may also lead to improved glucose metabolism through weight loss and improved insulin resistance. It is expected that a prospective study will be conducted in the future to assess the changes in each parameter after reducing circulating ANGPTL8 levels through therapeutic interventions.

### Limitations

There were several limitations associated with the present study. First, LPL values measured in this study were pre-heparin plasma LPL values, which may have led to the lack of a significant correlation seen between the LPL values and all the ANGPTL values. Second, a fasting blood sample was used, as opposed to postprandial blood, which may have yielded a different result. Third, we did not investigate the dynamics between ANGPTLs and dietary load, where the use of dietary loads with different carbohydrate, lipid, and protein contents may have caused variations in these proteins. Fourth, many patients monitored in this study were taking lipid-related drugs and hypoglycemic drugs, which could have influenced the results. The relationship between drugs and ANGPTL levels remains unclear, and this will have to be clarified via prospective studies. Fifth, it is unclear whether measured ANGPTL is free or present in a complex. IBL-ELISA ANGPTL3, 4, and 8 have never been compared to concentrations determined using mass spectrometry and there are no data on the correlation. It will be a future task to clarify which form of ANGPTL is measured by IBL-ELISA. Finally, the sample size used was small, and it is necessary to incorporate a larger sample to reduce selection bias and experimental error.

In summary, our study of patients with diabetes revealed that ANGPTL3, 4, and 8 are associated with high TG levels and low HDL-C levels. Although no direct interaction was observed among the plasma ANGPTL3, 4, and 8 concentrations, the group with high levels of both ANGPTL3 and ANGPTL8 showed the worst lipid profile. Among the ANGPTLs, ANGPTL8 was particularly important in determining the plasma TG levels; furthermore, ANGPTL8 may be associated with obesity and insulin resistance, as well as lipid metabolism. The results suggest interventions targeting ANGPTL8 as new treatment options for improving lipid and glucose metabolism in diabetes patients who need enhanced treatment. It is anticipated that an intervention method will be established to clinically decrease circulating ANGPTL8 levels for improving lipid and glucose metabolism.

## Supporting information

S1 File Data used for the analyses conducted in this study. (XLSX)
